# *In vitro* Assessment of Antiviral Effect of Natural Compounds on Porcine Epidemic Diarrhea Coronavirus

**DOI:** 10.3389/fvets.2021.652000

**Published:** 2021-03-29

**Authors:** Manuel Gómez-García, Héctor Puente, Héctor Argüello, Óscar Mencía-Ares, Pedro Rubio, Ana Carvajal

**Affiliations:** Department of Animal Health, Faculty of Veterinary Medicine, Universidad de León, León, Spain

**Keywords:** pig, organic acids, single essential oil compounds, antiviral activity, cytopathic effect

## Abstract

Organic acid and essential oils (EOs), well-known antimicrobials, could also possess antiviral activity, a characteristic which has not been completely addressed up to now. In this study, the effect of two organic acids (formic acid and sodium salt of coconut fatty acid distillates) and two single EO compounds (thymol and cinnamaldehye) was evaluated against porcine epidemic diarrhea virus (PEDV). The concentration used for each compound was established by cytotoxicity assays in Vero cells. The antiviral activity was then evaluated at three multiplicities of infection (MOIs) through visual cytopathic effect (CPE) evaluation and an alamarBlue assay as well as real-time reverse-transcription PCR (RT-qPCR) and viral titration of cell supernatants. Formic acid at at a dose of 1,200 ppm was the only compound which showed antiviral activity, with a weak reduction of CPE caused by PEDV. Through the alamarBlue fluorescence assay, we showed a significant anti-CPE effect of formic acid which could not be observed by using an inverted optical microscope. RT-qPCR and infectivity analysis also showed that formic acid significantly reduced viral RNA and viral titers in a PEDV MOI-dependent manner. Our results suggest that the antiviral activity of formic acid could be associated to its inhibitory effect on viral replication. Further studies are required to explore the anti-PEDV activity of formic acid under field conditions alone or together with other antiviral agents.

## Introduction

Organic acids and essential oils (EOs) are among the most effective alternatives to antibiotics (1), providing solutions to the antimicrobial resistance (AMR) problem (2). Compared to other cost-effective alternatives, organic acids and EOs have received particular attention due to their potential benefits to health and growth of the host (3, 4), associated to the modulation of gut microbiota and immune response (5–8). Apart from their known activity against bacteria, even with drug or multi-drug resistant profiles (9–12), studies using human viruses have shown that they may also have antiviral properties (13–16).

This activity needs further research in viral infections affecting other species. Among the viral enteric diseases of swine, porcine epidemic diarrhea (PED) is worth mentioning causing devastating economic losses for pig industry. It is a highly contagious non-zoonotic disease characterized by watery diarrhea and vomiting affecting pigs of all ages (17), particularly fatal in neonatal piglets due to malabsorption and dehydration (18). The etiological agent, porcine epidemic diarrhea virus (PEDV), is a coronavirus (CoV) which is an enveloped virus with a single-stranded positive sense RNA genome that infects small intestinal villous enterocytes (19). Current treatment relies on palliative strategies as there are no specific treatments or effective prophylactic vaccines which encourages the search for new alternatives for the treatment of PED (20, 21). Moreover, the use of PEDV as a model to study antiviral activity may also provide preliminary results on antiviral effect in other related CoVs (22). The pandemic outbreak of severe acute respiratory syndrome (SARS) CoV-2 has highlighted the importance of research on animal reservoirs of CoVs as well as on the prevention and control of interspecies transmission (23).

In this study, we evaluate the anti-PEDV effect of two organic acids, formic acid and sodium salt of coconut fatty acid distillates, and two single EO compounds, thymol and cinnamaldehyde, using three different multiplicities of infection (MOIs). The antiviral effect of these compounds was tested using cytopathic effect (CPE) evaluation and an alamarBlue assay as well as real-time reverse-transcription PCR (RT-qPCR) analysis and an infectivity assay of cell supernatants. Viral inactivation, attachment, entry and replication assays were also conducted to further characterize the antiviral mechanism of formic acid against PEDV.

## Materials and Methods

### Cell Line, Virus Propagation and Compounds Tested

Vero cells (African green monkey kidney cells) were cultured in Dulbecco's modified Eagle's medium (DMEM, Gibco) supplemented with 10% heat inactivated fetal bovine serum (FBS, Gibco) and 100 U/ml of penicillin, 100 mg/ml of streptomycin and 0.25 mg/ml of fungizone (Antibiotic-Antimycotic 100X, Gibco) at 37°C in 5% CO_2_ humidified incubator.

Cell-culture adapted PEDV CV777, provided by Dr. Hans Nauwynck (University of Ghent, Belgium), was propagated as previously described (24) in serum-free DMEM supplemented with 10 μg/μl trypsin (Trypsin 1:250, Gibco) and 0.3% tryptose phosphate broth (TPB, Sigma-Aldrich) (infection media). When cytopathic effect (CPE) reached 70–80% at about 18–24 h post-infection, the cells were subjected to three freeze-thaw cycles and the supernatant was collected, aliquoted and stored at −80°C until further use. Stock virus was assessed for viral titer following the Reed and Muench method and expressed as tissue culture infectious dose 50%/ml (TCID_50_/ml) (25).

Formic acid (purity 85%), sodium salt of coconut fatty acid distillates (67%), thymol (99%) and cinnamaldehyde (97%) were provided by Norel SA (Spain). EO compounds were diluted 1:1 in sterile propylene glycol (Sigma-Aldrich) while sodium salt of coconut fatty acid distillates, which was obtained as a powder compound, was resuspended in distilled water. Formic acid was provided as a liquid preparation.

### Cytotoxicity Assay

Cytotoxic effect of each compound was evaluated using the alamarBlue (resazurin) (Invitrogen) assay as described by Kumar and colleagues (26) with some modifications. Briefly, Vero cells were seeded at a density of 20,000 cells per well in a 96-well plate and let to reach sub-confluence. After three washes with sterile phosphate-buffered saline (PBS, pH 7.4), 200 μl of serum-free DMEM supplemented with different concentrations of each compound were added to each well and incubated at 37°C for 48 h. Cells were washed again and incubated for 2 h with 100 μl per well of DMEM supplemented with 10% alamarBlue. Fluorescence intensity (λ exc 530 nm and λ em 590 nm) was measured with a Sinergy HT microplate absorbance reader (Biotek Instruments). For each compound concentration, the relative cell viability rate (%) was calculated by comparing treated and control wells without treatment [(mean fluorescence of treated well/mean fluorescence of control well) × 100] and the 50% cytotoxicity concentration (CC_50_) was determined using regression analysis.

The concentration range tested for each compound was chosen considering usual inclusion rates of these compounds when used as feed additives (9) and included seven 2-fold serial dilutions: 4,800–75 ppm for formic acid thymol, 64–1 ppm for sodium salt of coconut fatty acid distillates and 2,400–37.5 ppm for cinnamaldehyde. Cytotoxic effect of propylene glycol (4,800–75 ppm) was also tested and each assay was carried out in triplicate.

### Evaluation of Antiviral Activity by Mean of Cytopathic Effect, RT-qPCR and Infectivity Assay

Antiviral activity of each compound was tested at a minimally toxic concentration which allowed for >75% cell viability. Vero cell monolayers grown in 96-well plates were washed three times and then treated with 100 μl per well of infection media (DMEM-Trypsin-TPB) containing each compound along with three 10-fold serial dilutions of PEDV (MOIs 0.50, 0.05 and 0.005). After 2 h incubation at 37°C in 5% CO_2_ humidified incubator, 100 μl of infection media with the same concentration of compound but without PEDV was added to each well. Plates were monitored for CPE at 48 h. In all, 16 wells were used for each condition tested.

After CPE evaluation at 48 h, the plates were subjected to three freeze-thaw cycles and the well contents of each eight identical replicas were harvested and mixed. A volume of 200 μl was used for RNA extraction using GeneMATRIX Viral RNA/DNA Purification Kit (EurX). RT-qPCR was carried out using a QuantStudio 1 thermal cycler (Applied Biosystems) and a commercial kit (EXOone PEDV, EXOPOL) provided with an endogenous control and amplifying a 191 bp product. Following the manufacturer's instructions, a standard curve was generated using 10-fold dilutions of the positive control standard (log_10_ viral RNA molecules/μl ranging between 6 and 1) to estimate the viral load. Each RNA sample was analyzed in duplicate.

Further investigation of antiviral activity was carried out by estimating viral titer in an infectivity assay. Briefly, 10-fold serial dilutions of cell supernatants in infection media were inoculated on Vero cell monolayers grown in 96-well plates and incubated at 37°C for 72 h. Cells were monitored for CPE and viral titer was estimated using the Reed and Muench method and expressed as TCID_50_/ml. Eight wells were used for each dilution and the assays were carried out in duplicate.

### Evaluation of Antiviral Activity Using an alamarBlue Assay

The alamarBlue assay was also used to elucidate the inhibitory effect on CPE caused by the compounds tested. For this purpose, eight wells of a 96-well plate were treated as previously described with 100 μl per well of infection media supplemented with a minimally toxic concentration of each compound and PEDV (MOI = 0.05). Non-infected control wells with 100 μl of infection media supplemented with each compound were also included. A standard curve prepared by inoculating 2-fold serial dilutions of PEDV in infection media (MOI ranging between 0.50 and 2.44 × 10^−4^) was used to confirm the efficacy of alamarBlue dye measuring the dose-dependent effect of PEDV on Vero cell viability.

After 48 h incubation, the supernatant of each well was removed and 100 μl per well of DMEM supplemented with 10% alamarBlue was added. Fluorescence was measured as previously described and the relative cell viability rate (%) was calculated by comparing infected and non-infected wells with product and infection media [(mean fluorescence of treated-PEDV infected wells/mean fluorescence of treated non-infected wells) × 100].

Positive (infected non-treated) and negative control wells (non-infected non-treated exposed to infection media and infected non-treated exposed to propylene glycol) were also included. Each assay was carried out in triplicate.

### Antiviral Mechanism of Formic Acid

Viral inactivation, attachment, entry and replication assays were conducted as previously described (20) to investigate the anti-PEDV mechanism of formic acid at a concentration of 1,200 ppm.

For viral inactivation assay, 1 ml of infection media with formic acid and PEDV (initial titer 5.5 log_10_ TCID_50_/ml) were incubated for 1 h at 4°C.Three 10-fold dilutions (1:10–1:1,000) were carried out and 100 μl per well were used to infect Vero cells for 48 h (MOI 0.50, 0.05, and 0.005). In order to conduct viral attachment assay, precooled cells were treated with 100 μl per well of infection media with formic acid and PEDV (MOI 0.50, 0.05, and 0.005) at 4°C for 2 h. After removing the supernatant by washing with cold PBS, Vero cells were again incubated with 100 μl per well of infection media at 37°C for 48 h. In order to test the effect on viral entry, 100 μl per well of the same three PEDV dilutions were added to Vero cells (MOI 0.50, 0.05, and 0.005) at 4°C for 2 h. Cells were then rinsed and incubated with formic acid at 37°C for 1 h followed using a new washing step and incubation at 37°C for 48 h. Finally, to evaluate the effect of formic acid on PEDV replication, cells were infected with 100 μl per well of PEDV at the same three MOIs for 2 h at 37°C. Then, 100 μl per well of infection media with formic acid were added and the culture was incubated for 48 h.

For each assay, controls with the same conditions but no formic acid were included and the inhibitory effects were determined by evaluating the CPE (*n* = 16) as well as infectivity and RT-qPCR analyses to estimate the viral titer and RNA molecules/μl, respectively, as previously described.

### Statistical Analysis

Relative cell viability rates obtained in the evaluation of antiviral activity using an alamarBlue assay as well as viral loads estimated by RT-qPCR and infectivity assays were tested for normality (Kolmogorov-Smirnov test) and statistical differences between experimental groups were evaluated using either ANOVA or Kruskal-Wallis test. The results of the inhibitory effect on CPE were analyzed by mean of the Yates' corrected Chi-Square test. These analyses were carried out with IBM SPSS Statistics version 26 at the 5% significance level.

## Results

### Cytotoxicity Assay

**Figure 1:**
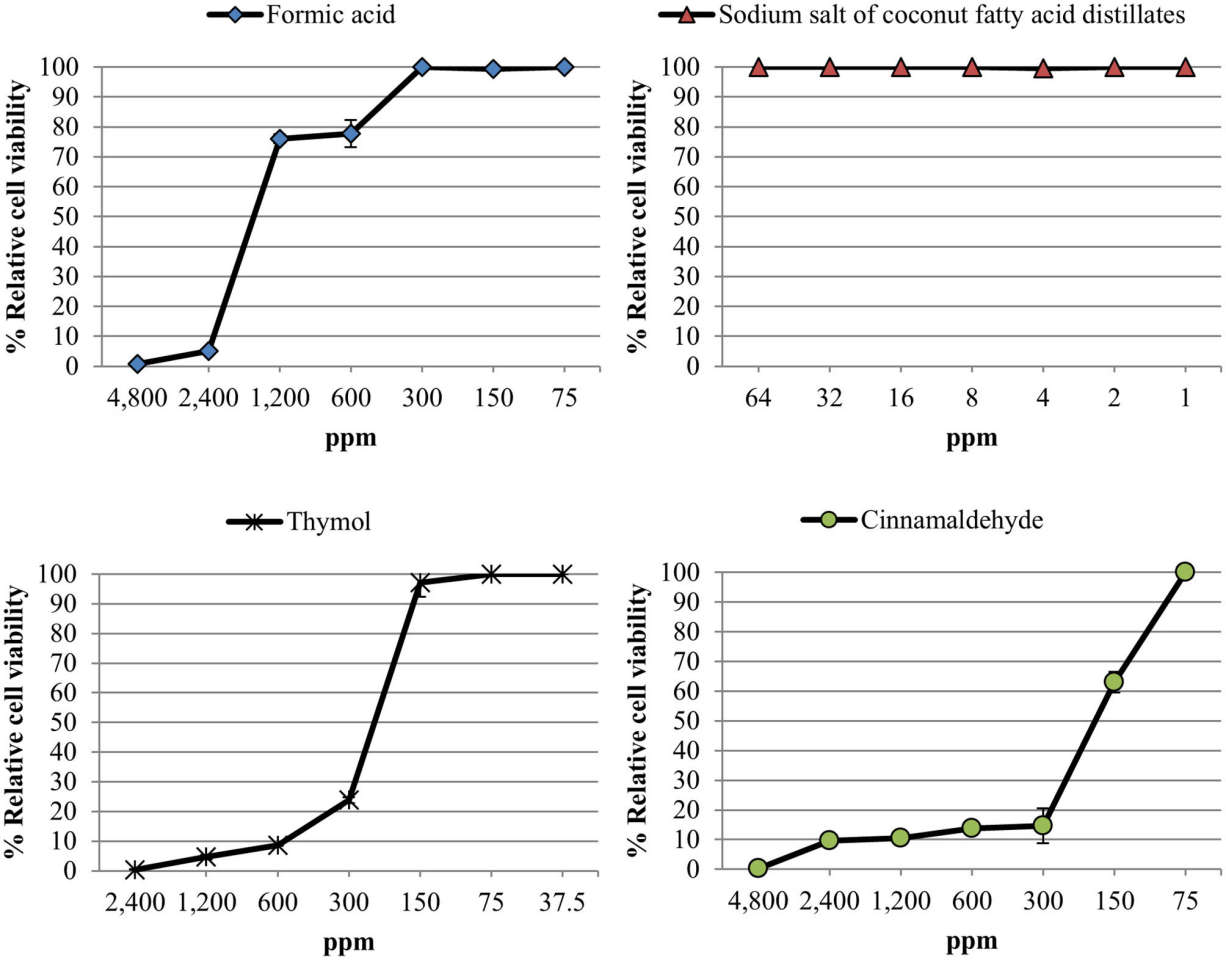
Relative cell viability rate [(mean fluorescence of treated well/mean fluorescence of control well) × 100] measured using an alamarBlue assay after exposure of Vero cell monolayers for 48 h to different concentrations of each compound tested. Data are expressed as mean of the three independent replicates ± standard deviations.

The relative cell viability after the exposure to different concentrations of the compounds tested is shown in Figure 1. All compounds showed a dose-response effect with the only exception of sodium salt of coconut fatty acid distillates which did not exhibit any cytotoxicity against Vero cells in the range of concentrations tested. The CC_50_ values were 1,586.9 ppm, 245.9 ppm and 203.5 ppm for formic acid, thymol and cinnamaldehyde, respectively.

### Evaluation of Antiviral Activity by Mean of Cytopathic Effect, RT-qPCR and Infectivity Assay

Due to high cytotoxicity showed by the compounds tested, a single concentration of each product was used to evaluate the anti-PEDV activity; 1,200 ppm for formic acid, 64 ppm for sodium salt of coconut fatty acid distillates, 150 ppm for thymol and 75 ppm for cinnamaldehyde.

**Figure 2:**
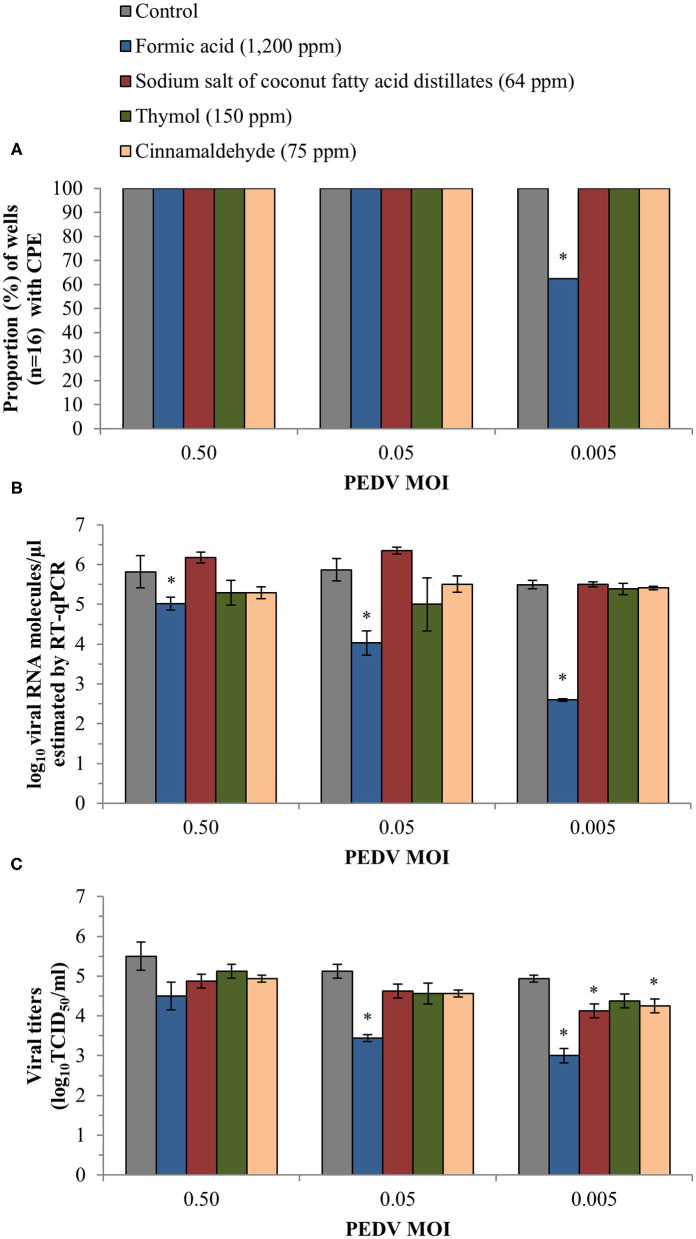
Anti-PEDV activity of two organic acids, formic acid and sodium salt of coconut fatty acid distillates, and two single EO compounds, thymol and cinnamaldehyde, determined in Vero cells infected with three different PEDV MOIs. **(A)** Proportion (%) of wells (*n* = 16) with CPE obtained after visual inspection of cells under an inverted light microscope. **(B)** Mean values ± standard deviations of log_10_ viral RNA molecules/μl estimated using RT-qPCR. **(C)** Mean values ± standard deviations of viral titer expressed as log_10_TCID_50_/ml and calculated following the Reed and Muench method. *Statistically significant differences with respect to controls (*p* < 0.05).

No antiviral activity was observed for any of the tested products using the CPE evaluation (100% CPE) with the only exception of formic acid at the lowest MOI (Figure 2A). The inclusion of 1,200 ppm of formic acid reduced CPE to 62.5% in the wells exposed to PEDV at a MOI of 0.005, a significant reduction compared to non-treated control wells (*p* < 0.05). No inhibitory effect on CPE was observed for propylene glycol.

Similarly, formic acid was the only compound which reduced viral load estimated by RT-qPCR in Vero cell supernatants. Significant differences (*p* < 0.05) were observed at the three different MOIs, with an average reduction of 0.97 ± 0.22, 2.04 ± 0.35 and 2.86 ± 0.49 log_10_ viral RNA molecules/μl as compared with the controls at 0.50, 0.05, and 0.005 MOI, respectively (Figure 2B). The RT-qPCR results also confirmed the absence of antiviral activity of propylene glycol (an average reduction estimated at the three MOIs of 0.07 ± 0.04 log_10_ viral RNA molecules/μl).

Finally, infectivity assay confirmed the reduction of fully infective viral particles formed when treated with formic acid (*p* < 0.05). We achieved a reduction of 1.13 ± 0.09 and 1.94 ± 0.18 log_10_TCID_50_/ml at 0.05 and 0.005 PEDV MOI, respectively (Figure 2C). For the rest of compounds, the viral titers were only reduced significantly (*p* < 0.05) compared to the control after exposure to sodium salt of coconut fatty acid distillates (an average reduction of 0.69 ± 0.18 log_10_TCID_50_/ml) and cinnamaldehyde (0.81 ± 0.18 log_10_TCID_50_/ml) at the lowest PEDV MOI tested.

### Evaluation of Antiviral Activity Using an alamarBlue Assay

**Figure 3:**
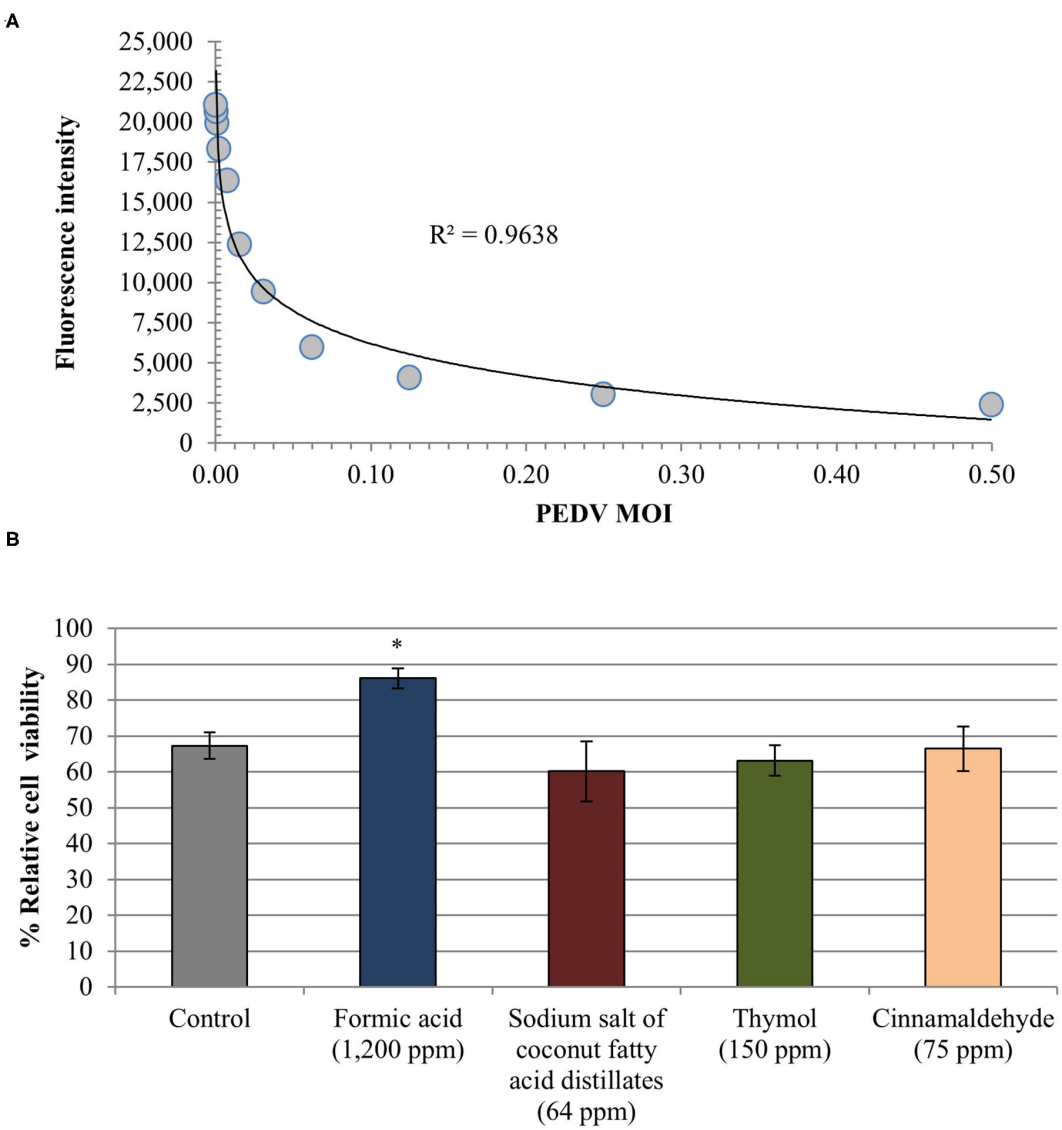
Anti-PEDV activity of two organic acids, formic acid and sodium salt of coconut fatty acid distillates, and two single EO compounds, thymol and cinnamaldehyde, determined in Vero cells by means of an alamarBlue assay for estimating cell viability. **(A)** Standard curve generated from fluorescence intensity associated with exposition to serial dilutions of PEDV (TCID_50_/cell). Linear trend line and *R*^2^ value obtained are shown. **(B)** Relative cell viability rate [(mean fluorescence of treated-PEDV infected wells/mean fluorescence of treated non-infected wells) × 100]. Data are expressed as mean of the independent replicates ± standard deviations. *Statistically significant differences with regard to controls (*p* < 0.05).

Linear regression analysis showed an almost perfect linearity (*R*^2^ = 0.964) of the standard curve generated validating the alamarBlue assay to quantitatively assess PEDV associated CPE (Figure 3A).

Formic acid reduced CPE associated to PEDV showing a higher relative cell viability rate (86.07% ± 2.85) as compared with PEDV infected non-treated wells (67.29% ± 3.75) (Figure 3B). Difference between both results reached statistical significance (*p* < 0.05). No effect was observed for the rest of compounds tested (average relative cell viability rate 63.22% ± 3.18) as well as for propylene glycol (65.72% ± 3.39). Finally, the alamarBlue assay confirmed that Vero cells viability was not affected by the infection media used.

### Antiviral Mechanism of Formic Acid

The evaluation of CPE showed that formic acid did not protect Vero cells from viral damage induced by PEDV neither as a consequence of viral inactivation nor interference with viral attachment or viral entry (100% CPE positive wells). However, an effect was observed in the viral replication at the lowest PEDV MOI evaluated (87.5% CPE positive wells).

**Figure 4:**
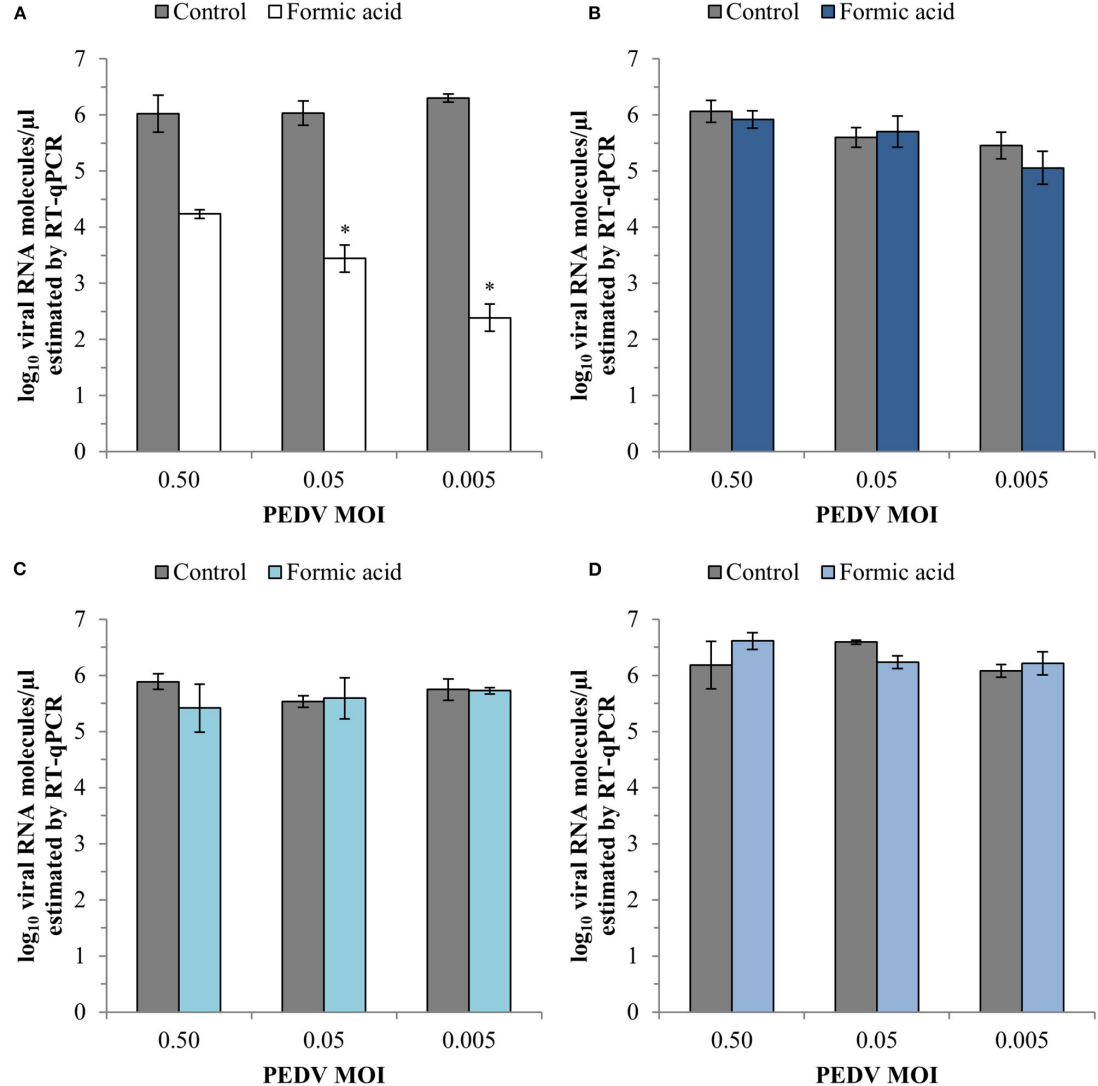
Effect of a treatment with 1,200 ppm of formic acid on the different steps of the infection cycle of PEDV: replication **(A)**, viral inactivation **(B)**, attachment **(C)** and entry **(D)**. Data are expressed as mean ± standard deviations of log_10_ viral RNA molecules/μl) estimated using RT-qPCR. *Statistically significant differences with regard to controls (*p* < 0.05).

RT-qPCR analyses showed a reduction of PEDV replication after formic acid treatment with average reductions of 1.79 ± 0.30, 2.59 ± 0.25 and 3.92 ± 0.18 log_10_ viral RNA molecules/μl in the cell supernatant at PEDV MOI of 0.50, 0.05, and 0.005, respectively (Figure 4A). A slight average reduction of 0.84 ± 0.32 log_10_ viral RNA molecules/μl was also observed in the viral inactivation assay at the lowest PEDV MOI tested (Figure 4B). No other effect of formic acid on the number of viral RNA molecules was shown in PEDV attachment (Figure 4C) and entry (Figure 4D).

**Figure 5:**
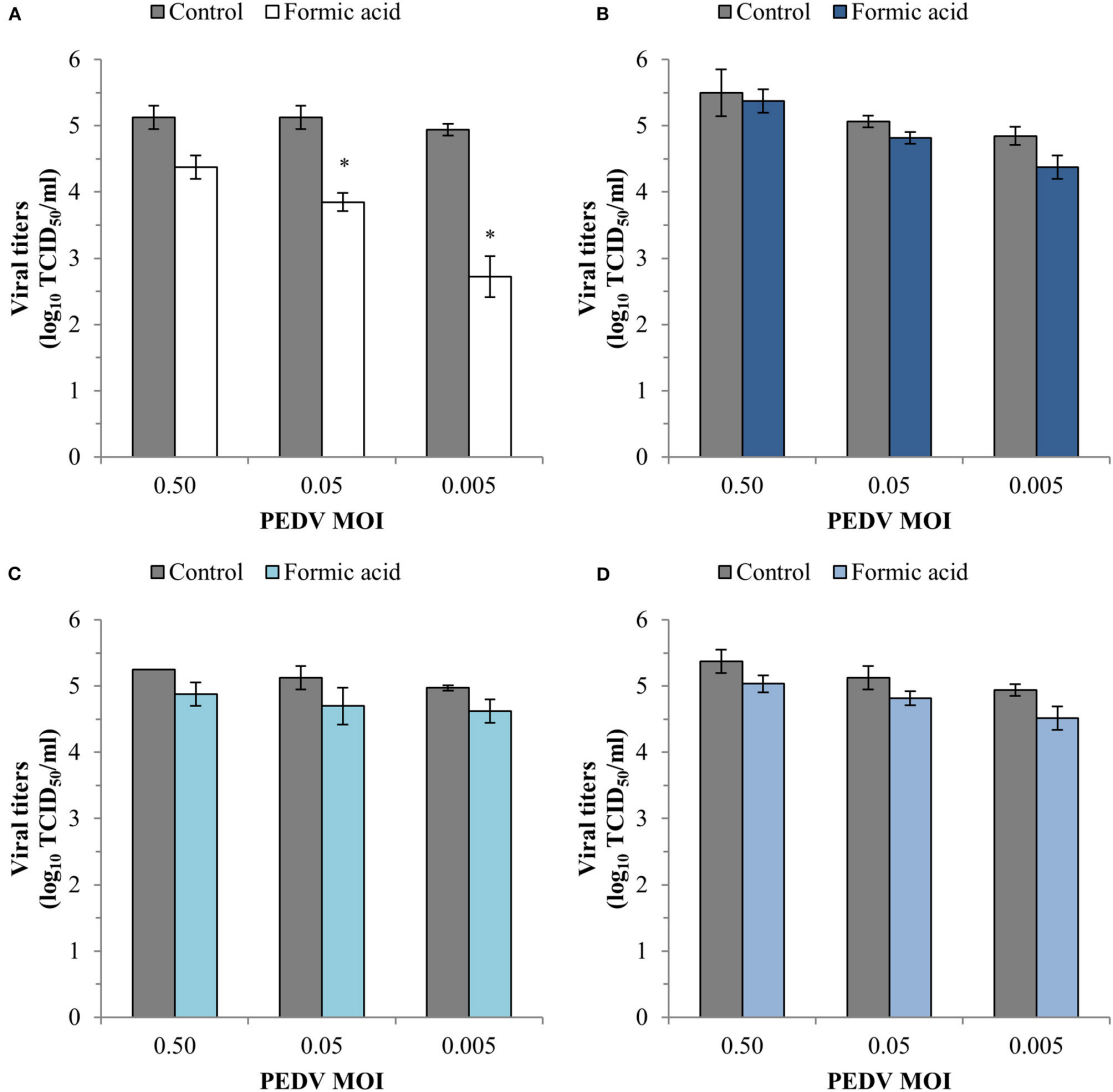
Effect of a treatment with 1,200 ppm of formic acid on the different steps of the infection cycle of PEDV: replication **(A)**, viral inactivation **(B)**, attachment **(C)** and entry **(D)**. Data are expressed as mean ± standard deviations of viral titer expressed as log_10_TCID_50_/ml estimated using an infectivity assay following the Reed and Muench method. *Statistically significant differences with regard to controls (*p* < 0.05).

The results of the infectivity assay also showed a significant reduction (*p* < 0.05) in the viral replication with viral titer reductions of 1.28 ± 0.14 and 2.22 ± 0.31 log_10_TCID_50_/ml at PEDV MOI of 0.05 and 0.005, respectively (Figure 5A). An average reduction of 0.34 ± 0.10 log_10_TCID_50_/ml for the three PEDV MOI tested was obtained in the other three steps of the infection cycle tested (Figures 5B–D).

## Discussion

The search for clinically effective antiviral chemotherapeutic products which can be used in the treatment of life-threatening animal diseases is one of the global veterinary priorities (23). The usefulness of the currently available methods for the control of PED has been increasingly questioned because of the emergence and re-emergence of PEDV on pig farms worldwide (20, 27, 28). Many scientific studies have recently focused on testing the antiviral activity of medicinal plants and natural compounds (29, 30). It is still not clear whether essential oils and organic acids, natural antimicrobials commonly used in animal nutrition (7, 31, 32), have any effect on porcine coronavirus or not.

Cytotoxicity assay on Vero cells, the most appropriate for *in vitro* propagation of PEDV (33), showed that the tested compounds had cytotoxic effect at target concentrations with the only exception of sodium salt of coconut fatty acid distillates. This result agrees with previous reports which have also shown a substantial decrease in cell proliferation at low concentrations of thymol and cinnamaldehyde (34). However, the lack of a dose-dependent effect on Vero cell viability of sodium salt of coconut fatty acid distillates disagree with the results of a previous research on the effect of lauric acid, its main fatty acid, on colon cancer cells (35). Therefore, it seems that cytotoxicity potential of these compounds could differ between cell lines. Undoubtedly, this cytotoxic effect has been a major constraint of our research limiting the assessment of the antiviral effect of these compounds to a single concentration.

Formic acid was the only compound which showed a consistent anti-PEDV effect. This activity was MOI dependent. The antiviral activity was only observed at the lowest PEDV MOI tested using CPE evaluation but it was confirmed using RT-qPCR at the three PEDV MOIs evaluated. A reduction in viral titer was also demonstrated for formic acid at the two lowest PEDV MOIs tested using an infectivity assay. The observed differences between CPE evaluation and RT-qPCR or infectivity assay results may suggest that formic acid does not protect Vero cells from viral damage induced by PEDV but could block viral RNA replication and the formation of new infective viral particles. Minor discrepancies in results compared to previous studies (36) can be attributed to definition of positive CPE, more accurate and strict in our research as the presence of a single area of syncytium by a fusion of cell membranes was sufficient to consider the well as CPE positive.

Previous studies have also pointed out the antiviral activity of formic acid against other viruses. For instance, Lee et al. (37) reported an inhibitory activity of formic acid against influenza A virus subtype H9N2. It is worth noting that the concentrations of formic acid used in this research were higher than the concentrations used here. However, the inhibitory effect of formic acid on PEDV was lower than that reported by previous studies with other compounds such as lithium chloride (LiCl) or polysaccharide from *Ginkgo biloba* exocarp (20, 21). Both compounds showed a striking anti-PEDV activity at low concentrations.

Otherwise, the lack of antiviral activity of the single EO compounds tested in our study against PEDV follows the lines of a previous study which evaluated two commercial feed additives based on benzoic acid and EO which had no antiviral activity in pig feed when tested individually (38).

Our results using an alamarBlue assay support the use of this viability dye to estimate the degree of CPE associated to PEDV infection in Vero cells, allowing the evaluation of the anti-PEDV activity of minimally cytotoxic concentrations of compounds as has already been described for other viruses causing discernible CPE (39, 40). The results obtained using this assay confirm the existence of a significant anti-CPE effect of formic acid which could not be previously observed by using an inverted optical microscope.

Detailed analyses of the effect of formic acid on the stages of the PEDV infection revealed that viral replication was the most affected with a PEDV MOI-dependent reduction in the number of viral RNA molecules estimated by RT-qPCR as well as in the viral titers estimated by an infectivity assay. Our results agree with previous researches which have also shown antiviral activity through the inhibition of the viral replication by organic acids and EOs (38, 41). Although the precise molecular mechanism of action has not been elucidated, the inhibition of viral RNA synthesis could be due to the effect of formic acid on enzymes involved in this process as has been shown for other organic acids and essential oils (42).

Finally, our results showed that formic acid did not exert any anti-PEDV activity in the early steps of viral cycle with the only exception of a slight effect of viral inactivation at the lowest PEDV MOI. By contrast, effect on PEDV-Vero cell interactions, as the virus attachment or entry to the cell, has been reported for other potential antiviral compounds by previous studies (20, 21). However, no effect on the survival of porcine deltacoronavirus (PDCoV) was observed in complete swine feed supplemented with a commercial acidifier containing formic acid (purity 61%) even when it was used at twice the manufacturer's recommended dose (92 μl to 5 g aliquots of complete feed) (43).

To sum up, our results suggest that PEDV remains viable and replicates in Vero cells in the presence of non-cytotoxic concentrations of sodium salt of coconut fatty acid distillates, thymol and cinnamaldehyde. However, formic acid at a dose of only 1,200 ppm can effectively reduce PEDV replication in Vero cells. The anti-coronavirus properties of formic acid could be useful in the control of this disease on swine farms and should be further investigated *in vivo*. It should also be investigated if the use of formic acid as feed additive might affect the effectiveness of feedback-induced infections currently used in the sows of PEDV infected farms to promote immunity and protect lactating piglets. Finally, we suggest that the anti-CPE activity evaluation of minimally cytotoxic concentrations of potential antiviral compounds using a cell viability marker should also be highly recommended to obtain precise results and to ensure that methodological aspects does not affect the quality of the data.

## Data Availability Statement

The raw data supporting the conclusions of this article will be made available by the authors, without undue reservation.

## Author Contributions

MG-G, HP, HA, PR, and AC conceived and designed the experiments, analyzed the data, and wrote and revised the manuscript. MG-G, HP, and ÓM-A performed the experiments. All authors contributed to the article and approved the submitted version.

## Conflict of Interest

The authors declare that the research was conducted in the absence of any commercial or financial relationships that could be construed as a potential conflict of interest.
